# Network-based group variable selection for detecting expression quantitative trait loci (eQTL)

**DOI:** 10.1186/1471-2105-12-269

**Published:** 2011-06-30

**Authors:** Weichen Wang, Xuegong Zhang

**Affiliations:** 1 Mathematics and Physics, School of Sciences, Tsinghua University, Beijing 100084, China; 2 MOE Key Laboratory of Bioinformatics/Bioinformatics Division, TNLIST, Beijing 10084, China; 3 Department of Automation, Tsinghua University, Beijing 100084, China

## Abstract

**Background:**

Analysis of expression quantitative trait loci (eQTL) aims to identify the genetic loci associated with the expression level of genes. Penalized regression with a proper penalty is suitable for the high-dimensional biological data. Its performance should be enhanced when we incorporate biological knowledge of gene expression network and linkage disequilibrium (LD) structure between loci in high-noise background.

**Results:**

We propose a network-based group variable selection (NGVS) method for QTL detection. Our method simultaneously maps highly correlated expression traits sharing the same biological function to marker sets formed by LD. By grouping markers, complex joint activity of multiple SNPs can be considered and the dimensionality of eQTL problem is reduced dramatically. In order to demonstrate the power and flexibility of our method, we used it to analyze two simulations and a mouse obesity and diabetes dataset. We considered the gene co-expression network, grouped markers into marker sets and treated the additive and dominant effect of each locus as a group: as a consequence, we were able to replicate results previously obtained on the mouse linkage dataset. Furthermore, we observed several possible sex-dependent loci and interactions of multiple SNPs.

**Conclusions:**

The proposed NGVS method is appropriate for problems with high-dimensional data and high-noise background. On eQTL problem it outperforms the classical Lasso method, which does not consider biological knowledge. Introduction of proper gene expression and loci correlation information makes detecting causal markers more accurate. With reasonable model settings, NGVS can lead to novel biological findings.

## Background

Genetic loci that affect the expression levels of mRNA are called expression quantitative trait loci (eQTL). Considering mRNA transcript abundance as a quantitative trait, the aim is to detect the associated genetic loci, which is the key to understanding the regulation network and disease phenotype. Thanks to the high-throughput and advanced sequencing technology, genome-wide linkage and association studies [1,2] have shown to be effective for finding causal gene loci for diseases in many species from yeast to human. The interested reader may find a detailed overview of the eQTL issues and some existing mapping methods in reviews [3,4].

The simplest mapping ideas are regression-based methods, but traditional methods have some disadvantages. Single QTL regression and the interval mapping method [5] tend to show too many associated loci and fail to take into account the complex interaction effects. While multiple-QTL approaches, such as the two-dimensional scan, consider such interactions, they are computationally expensive and have low statistical power due to multiple tests. These methods are based on the selection of a p-value threshold, thus if the threshold is not selected properly, high false positive rate occurs. Compared to multiple-QTL regression, variable selection methods seem to be more robust. Storey et al. [6] showed that the forward sequential search is more powerful than the exhaustive two-dimensional scan. However, since markers once selected cannot be removed from the model, the forward selection tends to select an excessive number of markers and only achieves local optimization. To overcome some weaknesses of the stepwise selection, Tibshirani proposed the Lasso penalized regression [7]. The Lasso method with L_1 _penalty produces interpretable models with some coefficients exactly 0. Two of its extensions are appealing. The Group Lasso or GLasso [8] on the one hand selects variables and reduces the dimensionality in a group fashion by applying L_2 _penalty to each group of variables. The elastic net method [9], on the other hand, by adding up the L_1 _and L_2 _penalties together, becomes ideal for " large p small n" problems with highly correlated data. However these excellent Lasso-based methods [10,11] are not designed for eQTL and more biological information should be incorporated to improve their performances. Therefore we aimed to develop a new penalty which can give more accurate selection of QTLs as well as allowing more flexibility of model setting for different biological prior knowledge.

Previous studies have demonstrated that incorporating biological information on genes with the same function would increase the accuracy of detection of hot spots [12-14]. Since the problem has small sample size, large noise and high dimensionality, we hope to borrow information from a gene expression network. It can be any kind of network: a network based on annotation system such as Gene Ontology (GO) [15] or KEGG [16], a clustering or co-expression network, a protein-protein interaction network etc. To add the network into our penalized regression framework, the network-constrained regularization method [17], an extension of the elastic net, is enlightening. The idea is simple: the difference between the coefficients of two connected genes on the network should be small. Pan applied this idea to his network-based method [12], and the results clearly demonstrate the advantage of methods utilizing gene networks.

Besides the gene expression network, correlations between markers or linkage disequilibrium (LD) structure are very informative. The true causal SNPs are rarely genotyped but may be in LD with near markers. In addition the epistatic effects among different SNPs can be very large, with each individual SNP' s effect very low. In these cases we need complex models rather than linear ones to describe the LD structure. Wu et al. proposed to group SNPs into SNP sets based on LD structure in the association study, and then test the joint effect of each SNP set [18]. We applied this idea to our regression framework and selected the markers at the group level just like the GLasso [8]. As a result, our method: (1) has more power to detect significant loci, (2) allows us to consider the complex joint activity of SNPs within each marker set, (3) better captures untyped causal SNPs, (4) reduces the dimensionality of the problem dramatically, and (5) may be combined with other existing low-dimensional selection methods for further study. It is also worthwhile to point out that by forming marker sets, we are able to consider the additive and dominant effects of one locus as a group. Naturally, the effects of the three different kinds of genotypes (AA, Aa, aa) of one SNP should be represented by two dummy variables, which exist or not at the same time. In addition, once we group some markers into a set, covariate models and different epistatic models can be constructed within the set, providing additional information to understand the true biological regulatory mechanism.

## Methods

### Network-based group variable selection

Suppose that the dataset has n samples and p markers. We have G quantitative gene traits ***Y**_**1**_, **Y**_**2**_,..., **Y**_**G**_, where ***Y***_**g **_= (y_*1g*_, y_*2g*_,..., y_*ng*_)^*T*^, g = 1,..., G and we combine them to form the entire gene expression vector . The p markers can be divided into J blocks describing the J marker sets, where the j^th ^marker set for the i^th ^individual is **x**_***j, i ***_= (x_*j1, i*_, x_*j2, i*_,..., j_*pj, i*_), j = 1,2,..., J, p = p_*1*_+p_*2*_+ · · · +p_*J *_being the total marker number. Then the marker data matrix is ***X***_***M ***_= (***X***_***1***_, ***X***_***2***_,..., ***X***_***J***_), where ***X***_***j ***_= (***x***_***j,1***_^*T*^, ***x***_***j,2***_^*T*^,... ***x****_***j, n***_^*T*^)^*T*^. We combine them to get the entire marker data matrix ***X ***= diag (***X***_***M***_, ***X***_***M***_,..., ***X***_***M***_). Note that all the *G *traits come from the same genotype data and the marker data matrix is the same for all traits. We then regress all gene traits ***Y ***on the marker data ***X***. After the location and scale transformation, we can assume that the regressors are standardized and each response is centered, obviating the need to consider the intercepts.

Let' s consider a network that is represented by a graph with *E *edges and *G *vertices. Each Vertex represents a trait, an edge *u~v *indicates that gene trait *u *and *v *are linked on the network. Let' s define the degree *d_u _*of the vertex *u *as the total number of edges linked to *u*; and suppose *d_u _> 0 *for each *u*. To describe the structure of the network, we use matrix ***L ***similar to [17]. The *p *by *p *block element ***L**(u, v) *of ***L ***is defined as:

where ***I***_***p ***_is the identity matrix of order *p*. Since ***L ***is always non-negative definite, it can be decomposed as ***L ***= ***SS***^*T*^, where ***S***_*Gp × Ep *_is the matrix in which, taking every *p *by *p *matrix as one block, the block rows are indexed by the vertices and block columns are indexed by the edges of the graph such that each block column corresponding to an edge *u~v *has an entry  in the row corresponding to *u*, an entry  in the row corresponding to *v *and zero elsewhere.

For any pair of fixed non-negative tuning parameters *λ_1 _*and *λ_2_*, we define our network-based group variable selection (NGVS) criterion:(1)

where ***β ***= (***β***_***1***_^*T*^,..., ***β***_***G***_^*T*^)^*T*^, ***β***_***g ***_= (***β***_***1, g***_^*T*^,... ***β***_***J, g***_^*T*^)^*T*^, ***β***_***j, g ***_= (*β*_***j1, g ***_*β*_***j2, g***_,...,*β*_***jpj, g***_)^*T *^and the norm is L_2 _norm. The first term is the sum of squared errors. The second term is identical to the GLasso penalty only with an additional sum over gene traits and is used for selecting marker sets. The weights  ensure that the penalty term is of the order of the parameter number of each group. The third term can be written as

where Σ_u~v _denotes the sum over all unordered pairs (*u*, *v*) for which *u *and *v *are linked on the network. The third term actually reveals the assumption that genes which are highly correlated and truly regulated by the same QTLs tend to have the same effect. The NGVS estimator  is the minimizer of Equation (1), i.e.(2)

The following lemma shows that minimizing our NGVS criterion is equivalent to solving a GLasso-type optimization problem, thus can be computed by some efficient existing algorithms.

*LEMMA 1. Given dataset *(***Y***, ***X***) *and two fixed tuning parameters (λ*_*1*_, *λ*_*2*_), *define an artificial dataset *(Y*, X*) *by*

*where ****S ****is the decomposition of ****L***. *Let **and **Then the NGVS criterion can be written as*

*Let **be the solution to the above GLasso minimization problem; then the solution to (2) is *

Following Zou and Hastie [9], the NGVS estimator should be adjusted by a factor of 1+λ_2 _due to the possible bias of double shrinkage. From Lemma 1, the NGVS problem can be reformulated as an equivalent GLasso problem by augmenting the dataset from *Gn *to *Gn+Ep*. Therefore, when doing variable selection, this model can select all *Gp *variables if *Gn+Ep > Gp*. GLasso can only select at most *Gn *variables before it saturates. By choosing a network with the total number of edges bigger than *G(p-n)/p*, even when *n *is much smaller than *p*, we can overcome the limitation. This can be easily accomplished by using a smaller correlation threshold or making the network sufficiently big.

*LEMMA 2*. *is determined by Equation (2). Assume that gene u and v are only linked with each other on the network and the corresponding response vectors are equal, i.e*. ***Y***_*u *_= ***Y***_*v*_, then *for any λ*_*2 *_>*0 where **is the estimated coefficients for gene g*.

Lemma 2 is true since the penalty is a strictly convex function with *λ_2 _*> 0. This lemma shows the grouping effect of NGVS, which means that coefficients corresponding to highly correlated gene traits on the network tend to be the same. Therefore, our method can borrow information from traits with the same underlying function.

### Block co-ordinate gradient descent algorithm

Some algorithms are available for solving the GLasso problem. Yuan and Lin provided an iterative algorithm [8], but they realized that the computation burden explodes dramatically as the number of regressors increases. They also proved that GLars and GGarrote are not suitable for this problem, which are both the group forms of the Lars algorithm [19]. To handle " large p small n" problems efficiently, Meier et al. developed their block co-ordinate gradient descent (BCGD) algorithm [20]. The method can be applied to any generalized linear model where Y has an exponential family distribution.

The key idea of BCGD method is to combine a quadratic approximation of the log-likelihood with an additional line search. We first pick a zero vector as the initial coefficient vector, denoting no groups have been selected. Then by approximating the nonlinear log-likelihood by a second-order Taylor expansion at β of the last iteration and replacing the Hessian of the log-likelihood by a proper matrix, the minimization direction is found and β is updated by a point of that direction. Thus, either a new group will be selected, or the coefficients of previously selected groups will be changed slightly. The algorithm is fast in computing a whole range of solutions given sufficiently small grid on penalization parameters and then generating the selection order. The algorithm is available in the R-package grplasso.

### Marker sets and gene expression networks

Biological information incorporated by our proposed NGVS method mainly include gene expression network and loci correlation, that is, the way to form marker sets. Proper grouping of markers based on the prior knowledge can increase the power to detect causal SNPs, while bad division of marker sets may probably harm the results since the unlinked loci may dilute the effect of causal loci. Basically, all grouping ideas can be divided into three categories: LD-based, knowledge-based, and convenience-based. In GWAS, grouping SNPs in or near a gene is an ideal method; while in linkage analysis, because of the limited number of markers, grouping highly correlated markers produces good results. Genes that are located within a gene pathway often share biological functions and could be considered as a group. A more detailed analysis about how marker sets are formed can be found in [18].

The gene expression network, if properly set, could come from any source such as GO or KEGG pathways [15,16], clustering or co-expression network, PPI network etc. One way of constructing the network is, as we did in the real data analysis, to first identify a group of gene traits which share the same biological function by means of an external database, then to construct a co-expression network by a reasonable cutoff for the correlations between trait pairs using the same or a second dataset. The cutoff should be chosen such that the network satisfies the inequality E>G(p-n)/p as discussed above with the degree of each gene trait bigger than 0. The network provides a good performance in real data analysis.

### Selection orders and tuning parameters

With our NGVS method, for each fixed λ_2_, we are able to generate a selection order of the marker sets for a wide range of choices of λ_1_. We call this the big scale selection order as it describes the ranking of importance for the groups of markers. Once the relative importance of marker sets is established, further selection order of markers within each marker set can be produced by various existing methods. We call this the small scale selection order. To generate the final selection order of individual QTLs, we try to combine the two different scales together. Hence we need to go over a three-stage procedure: firstly getting the big scale selection order with NGVS; then finding the small scale selection order by any method suitable for low-dimensional selection; finally, combining the two selection orders together according to the three criteria discussed below.

In the first stage, we face a " large p small n" variable selection problem. Though we can select the optimal parameters by Cross-validation or some kind of C_p _or GCV criterion, it is time-consuming for two-dimensional tuning parameters. Based on our experiments, when considering big scale ranking, the results are quite stable against different *λ_2_*'s. So we use *λ_2 _*= 10 in our analysis and for this given *λ_2_*, we let *λ_1 _*vary over a wide range of grid to give the big scale selection order. The step size of *λ_1 _*should be small enough to guarantee that at most one new marker set is selected at a time. In the second stage, we have reduced the problem to be a " large p small n" variable selection problem. So all methods designed for low-dimensional ranking should be suitable, though we prefer to use the GLasso, which can select additive and dominant effects as one group. The small scale selection orders within each marker set are obtained without considering the loci structure and the co-expression network. This is because small scale differences of each gene trait are allowed. Furthermore, for a low-dimensional problem, simple selection methods are accurate enough to detect QTLs and considering the network may lead to bias (see the first simulation). Once the selection orders in two scales are ready, we apply three criteria to combine them in the final stage. Firstly, the most significant loci in each marker set are ranked according to the big scale selection order with NGVS; secondly, loci within each marker set are ranked according to the small scale selection order with GLasso; thirdly, when several loci satisfy the first two criteria, the locus with the smallest p-value for single QTL regression should be selected ahead of the others. Here, single QTL regression means assessing the significance of each individual SNP using the likelihood ratio test. The final selection order of all the markers will be determined uniquely by these three criteria. The final order is a combination of macro-order based on prior biological information, micro-order within each small group and single QTL p-values ranking. If we want to detect the causal QTLs of a certain trait, we can identify as significant the first desired number of loci in the final selection order.

**Figure 1:**
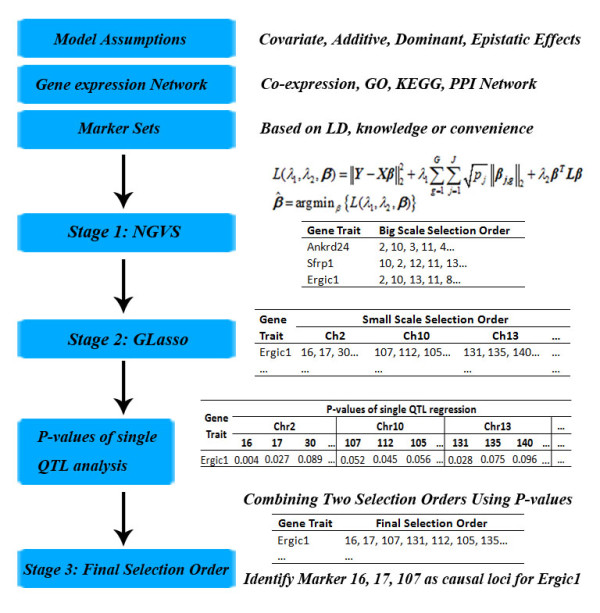
**The selection orders and the three-stage procedure**. In stage 1, we get the big scale selection order by NGVS. NGVS incorporates the prior knowledge of LD structure and gene expression network with the flexibility of model setting. In stage 2, we find the small scale selection orders within each marker set by GLasso. In stage 3 we combine the two selection orders together using P-values of single QTL regression and the three criteria discussed. Results in the picture were obtained from the adjacent interaction two-effect model (model assumption), full co-expression network of gene *Ankrd24*, *Sfrp1 *and *Ergic1 *(gene expression network) and marker sets formation by chromosomes (marker sets).

However, if we care more about general findings for a class of gene traits, we should pick out, according to the big scale order, marker sets which are identified as significant in most traits, then form the final selection orders and make conclusions only using markers in these identified marker sets. The whole process is shown in Figure 1.

### Assumptions for covariate, additive, dominant and epistatic effects

Four assumptions of our method are listed here: (1) the distribution of the error term is normal; (2) markers that are in high LD regions together reflect more information than one single marker; (3) highly correlated traits tend to be determined by the same loci; (4) covariate, additive, dominant and epistatic effects should be assumed based on some prior knowledge. Covariates such as age and sex sometimes are quite influential for gene expressions, hence cannot be ignored. If one covariate is significant, typically, we add it into the regression model as one additional group, together with the interactions of the covariate with markers. As for the additive and dominant effect, we assume that one SNP has only additive effect or both effects. Models containing two effects can provide more accurate QTL detection than the additive models as is shown by our results.

Epistatic effect refers to the interaction of multiple genetic variants. However, how this joint activity really happens is hard to detect. We propose two possible ways to describe epistasis - kernel model and near interaction model. If we focus on only the *g^th ^*gene trait and its corresponding *j^th ^*marker set, then by representer theorem [21], the relationship function *h_j, g _*of markers within the marker set can be written as:

where *K(·,·) *is some kernel function defining the epistatic relationship. Thus we extend the original regression model for the *g*^th ^trait **Y**_g _= **X_0_β**_g_+**ε**_g _to be:

For example, the linear kernel defines a linear model just as **Y**_g _= **X_0_β**_g_+**ε**_g_, but with an increased degree of freedom when *p_j_<n*. Note that kernel function is applied to each marker set. Thus with the same division of marker sets, we can treat kernels as *J×n *correlated new variables and get the big scale selection order as before. Essentially, kernel function projects nonlinear relationship into a higher dimensional space and the regression is then modelled linearly in the new space. The kernel can also be intuitively interpreted as the measure of similarity between two individuals. After this representation, we need only to introduce different kernels to specify the epistatic model and here we present five kernels we will use in our analysis.

(1) Linear Kernel: 

(2) Polynomial Kernel: 

(3) Gaussian Kernel: 

(4) Identical-by-state (IBS) Kernel: 

(5) Weighted IBS (WIBS) Kernel: 

where  and *q_jk _*is the minor allele frequency (MAF) for the *k*^th ^marker in the *j*^th ^group. The first kernel is linear while the second adds the interactions of SNPs to the *q*^th ^order. The next two kernels basically give various ways to measure the distance between two individuals. And the WIBS kernel is a correction of IBS for the rare alleles because they are usually more informative than common alleles. [18] offers more detailed explanation about these kernels and how to select a proper kernel.

Despite the many choices of kernels, it can be advantageous to switch to traditional two-locus interactions because, if showen to be significant, the biological interpretation is easier. We can extend each marker set to contain all the interactions between SNP pairs in the set and treat each of them as one new variable. However, when the number of SNPs in one marker set is large, we tend to only add near interactions. In high LD regions, we may consider interactions of two loci a little farther apart, while in low LD regions or with SNPs not densely genotyped, interactions of adjacent markers are enough. We will consider the adjacent-locus interaction model together with different kernel models in our analysis.

## Results and Discussion

To evaluate the performance of our proposed NGVS method, we simulated two models: the first to illustrate the advantages and disadvantages of the method in a low-dimensional setting; the second to mimic the high dimensional real problem.

### "Large n small p" simulation

First, we generated seven latent variables *Z*_*1*_,..., *Z*_*7 *_denoting genotypes according to a centered multivariate normal distribution whose covariances were *Cov(Z_1_, Z _2_) = 0.8 *and *Cov(Z_i_, Z_j _) = 0.4^|i-j| ^*for *i<j *and *(i, j)≠(1,2)*. Then *Z_i _*was trichotomized as -1, 1, 0 if it is smaller than Φ^-1^(1/4), larger than Φ^-1^(3/4) or in between respectively, representing genotype aa, AA, Aa. And we considered the linear model:

where *Z_1, i_, Z_2, i_, Z_3, i _*were one realization of *Z_1_, Z_2_, Z_3 _*for individual *i*; *Y_i, g_'s *denoting the gene expressions determined by additive and dominant effects of loci 1, 2, 3, which were modelled as two dummy variables - one for genotype AA and one for Aa; the expression network of two linked genes was also considered, i.e. *g = 1, 2*; *r_i, j, g_~U(0.9,1.1), i = 1,2,3, j = 1,2 *was a scaling factor used to perturb the effect size of the marker on trait *g*; finally ***ε***_***i***_= *(ε*_*i,1*_, *ε*_*i,2*_*) ~ N(0, Σ) *where *Σ*_*ij *_= *0.5σ*_*i*_*σ*_*j *_for *i *≠ *j*, *Σ*_*ii*_= *σ*_*i*_^*2 *^and *Σ *was determined by our choice of the signal-to-noise ratio (SNR), which is defined as the expected value of the mean square over the variance of expressions. Two different SNRs of 1 and 5 and two different ways to form the marker sets were tried. For each case, 50 simulated datasets were generated independently to calculate sensitivity and specificity.

The first way to construct marker sets is that *Z_1 _*and *Z_2 _*or four corresponding dummy variables are grouped into one marker set; and the two dummy variables for each of the other 5 loci build up the other 5 marker sets. This division for marker sets assumes that we have some prior knowledge about the higher correlation between *Z_1 _*and *Z_2_*.We call this marker set formation with SNR = 1 and SNR = 5 model 1 and 2 respectively. The second way to construct sets is to group *Z_1_, Z_2 _*and *Z_4 _*or six corresponding dummy variables into the first marker set; *Z_3 _*and *Z_5 _*are grouped into the second one and *Z_6_*, *Z_7 _*in the third one. This division represents a bad set formation because every significant locus is tangled with some insignificant one. We call this division with SNR = 1 and SNR = 5 model 3 and 4 respectively.

**Figure 2:**
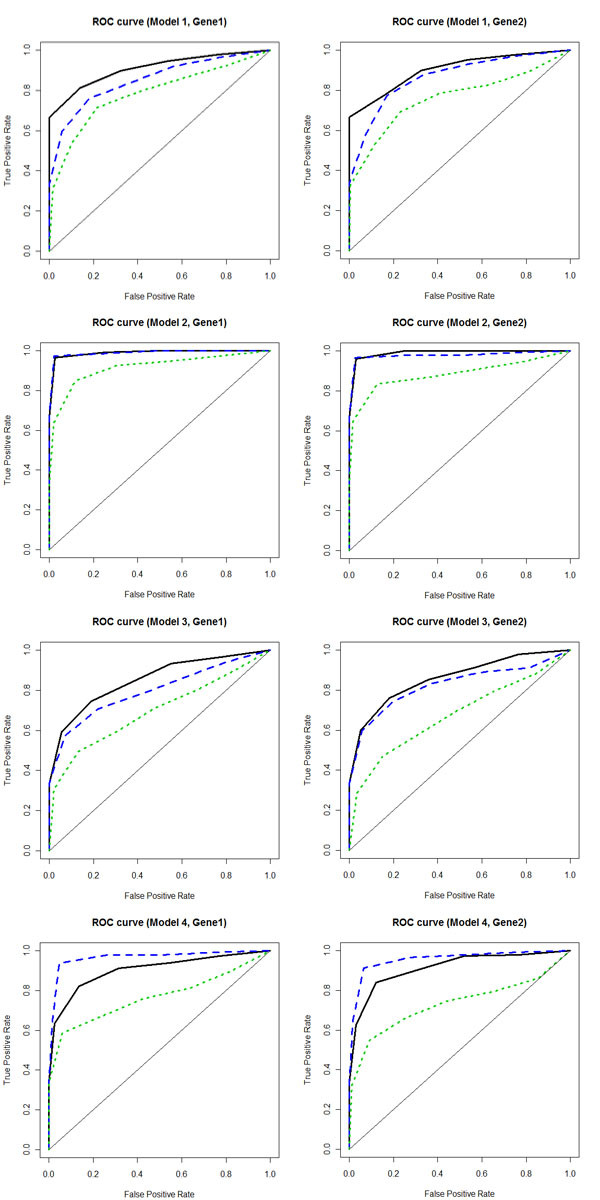
**Comparison of ROC curves of NGVS, GLasso and Lasso**. Black solid line: NGVS; Blue dashed line: Glasso; Green dotted line: Lasso. All three methods were tried in four models. Model 1 and 2, with SNR = 1 and 5 respectively, used proper division of marker sets. Model 3 and 4, with SNR = 1 and 5 respectively, used bad division of marker sets.

We compared three methods: (1) our proposed NGVS which combines gene expression network and loci structure; (2) GLasso which scans that information and merely selects additive and dominant effects simultaneously trait by trait; (3) the traditional Lasso method which only considers additive model. In NGVS, the selection order was constructed as illustrated above. And in the other two methods, selection orders were obtained by applying a wide range of different tuning parameters. Sensitivity and specificity of identifying the first three loci in the final selection order as significant loci are reported in Table 1. And the ROC curves corresponding to the 4 models are shown in Figure 2.

**Table 1:** Sensitivity and Specificity of the " large n small p" simulation

		Sensitivity			Specificity		
	
Model	Gene	NGVS	GLasso	Lasso	NGVS	GLasso	Lasso
1	1	0.81 (0.17)	0.76 (0.21)	0.71 (0.24)	0.86 (0.13)	0.82 (0.16)	0.79 (0.18)
	2	0.78 (0.16)	0.77 (0.21)	0.69 (0.23)	0.84 (0.12)	0.83 (0.16)	0.77 (0.17)
2	1	0.97 (0.10)	0.97 (0.09)	0.85 (0.18)	0.98 (0.08)	0.98 (0.07)	0.89 (0.14)
	2	0.96 (0.11)	0.97 (0.10)	0.83 (0.18)	0.97 (0.08)	0.98 (0.08)	0.88 (0.14)
3	1	0.75 (0.21)	0.71 (0.20)	0.59 (0.22)	0.81 (0.16)	0.78 (0.15)	0.70 (0.16)
	2	0.76 (0.18)	0.74 (0.22)	0.58 (0.19)	0.82 (0.13)	0.81 (0.16)	0.69 (0.14)
4	1	0.82 (0.20)	0.94 (0.13)	0.67 (0.17)	0.87 (0.15)	0.96 (0.10)	0.76 (0.13)
	2	0.84 (0.18)	0.91 (0.16)	0.66 (0.16)	0.88 (0.14)	0.94 (0.12)	0.75 (0.12)

NGVS: the network-based group variable selection method; GLasso: the group lasso without gene network and loci structure; Lasso: only considering additive effect of each locus. Sensitivity and specificity are calculated based on 50 simulations, with standard errors reported in parentheses.

From Table 1 and Figure 2, it is clear that our method is more powerful than Lasso in all of the four models. This is because Lasso only considers the additive effect of each locus when the underlying mechanism truly contains two effects. Methods selecting two effects in a group manner such as the Glasso and the NGVS perform better. In model 2 and 4, where SNR = 5 meaning that we have sufficient information for detecting QTLs accurately, Glasso provides fairly good results. If the signal is strong enough, adding improper loci grouping and gene network may increase uncertainty, thus impair sensitivity. In model 2 where we have proper marker set division, no significant difference in AUC between NGVS and GLasso is discovered; while in model 4 with bad maker sets formation, AUC of NGVS is reduced. However, in model 1 and 3, where background noise is important such that we do not have enough knowledge to select significant loci individually, by combining markers into marker sets, our NGVS method is more powerful than Glasso. Even when the marker sets formation does not reflect the true LD structure (model 3), adding network information and loci structure still increases sensitivity. In sum, if high-noise background is present, the information each QTL provides is not enough. We are able to obtain more powerful and convincing results by combining QTLs into marker sets and combining highly correlated gene traits into a network, then putting the information into our proposed NGVS framework.

### "Large p small n" simulation

In the second simulation, we considered a simulated dataset including 60 samples, each with the data of 200 markers, or 400 dummy variables, and 5 gene traits, to mimic a real linkage analysis. We first generated genotypes denoted by *Z*_1_,..., *Z*_*200 *_according to a centered multivariate normal distribution. The covariances are set to decrease as the distance between markers increases and 0 when the markers are more than 10 markers apart. Like before, *Z_i_'s *were trichotomized as -1, 0, 1. We considered the following model:

where *Z_3, i_, Z_4, i_, Z_13, i_, Z_27, i _*belong to three different marker sets *1,2,3*; *r_i, j, g_, ε_i, g _*are defined as before and SNR = 5. Twenty simulated datasets were generated independently to calculate True Positives (TPs) and False Positives (FPs). Our main aim here is to find out the causal markers *Z_3_, Z_4_, Z_13 _*and *Z_27_*.

We applied our three-stage selection procedure. We first selected significant marker sets using our NGVS method based on the additive-and-dominant-effect model; then GLasso was used to find the causal markers within each marker set; finally we decided the final selection order for all the loci. The full network of the 5 gene traits considered here obviously satisfies our requirement *E>G(p-n)/p*. For the marker set formation, *r *markers *rj-r+1,..., rj *were grouped to form the *j*^th ^marker set, *j = 1,...,(200/r) *and *r = 1, 2, 5, 10*. Note that *r = 1 *means we do not actually have a marker set and select the QTLs individually. In brief, we can either choose to use the gene expression network or not and choose among 4 different marker set formations - a total of 8 possibilities. When the network was utilized, we applied our proposed NGVS method under the 4 different marker set formations. If the network was not taken into account, we considered each individual gene trait respectively, but still maintained the 4 different marker set structures. We call this single trait selection, which means loci structure was taken into account but the QTLs were selected for each trait individually. In single trait selection, if *r = 1*, it is just GLasso used to select two dummy variables of one locus as a group for each trait. Besides the 8 possibilities, we also compared the selection orders of the single QTL regression and the Lasso. Single QTL regression assumed linear simple regression and tested whether the slope was significantly different from zero by likelihood ratio test. The selection order came from the ranking of p-values. Note that our method is a combination of the big scale NGVS, the small scale GLasso and the p-values coming from single QTL regression as the adhesive tool of the two scales.

**Figure 3:**
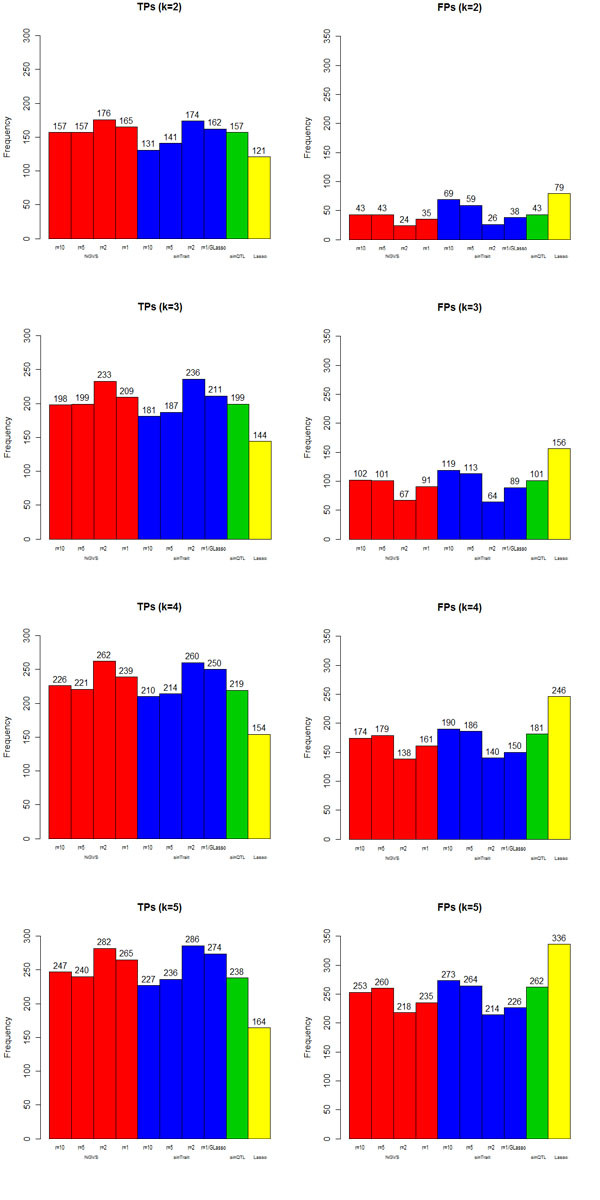
**TPs and FPs of NGVS, single trait selection, GLasso, single QTL regression and Lasso**. Red blocks: NGVS with marker set formation r = 10, 5, 2, 1 from left to right; Blue blocks: single trait selection with r = 10, 5, 2, 1 from left to right; single trait selection with r = 1 is just GLasso; Green block: single QTL regression; Yellow block: Lasso. The total true and false positives of the 5 gene traits of the 20 simulated datasets, if we identify the first k = 2, 3, 4, 5 QTLs as significant, were calculated based on the final selection order of each method.

The total TPs and FPs of the 5 gene traits of the 20 simulated datasets if we identified the first k loci in the final selection orders as significant are shown in Figure 3. Our NGVS method with marker set formation *r = 2 *performed the best. The effect of the scale of marker sets is shown through the first 4 columns of Figure 3. The histogram of first 4 columns is slightly U-shaped, which suggests that considering LD structure properly can increase power, but including too many non-causal markers will dilute the effect of causal ones. The proper way to form marker set as we discussed before should be decided based on prior knowledge and LD correlation. Single trait selection with proper marker set scale gives almost the same result with NGVS. However, under marker set formation *r = 10 *and *r = 5*, the 5 gene network protected NGVS from suffering the power decrease generated by containing too many unlinked loci into marker sets. Borrowing information among correlated gene traits reduces the risk of using improper division of marker sets. Comparing NGVS with GLasso, we conclude that adding a proper loci grouping and gene expression network indeed can improve the performance. Single QTL regression and the GLasso are probably useful for coarse ranking, but not good enough. In addition, the comparison between NGVS with Lasso illustrated the importance of considering the additive and dominant effects together. Methods lacking the description of the latent two-effect mechanism can only select markers of strong effect. In summary, when the sample size is smaller than the marker size, our method considering two-effect model, marker sets and correlated traits together can discover more casual QTLs of moderate effect and give more true positives.

### Real linkage data analysis

We analyzed a published mouse linkage dataset deposited at the gene expression omnibus (GEO) by Lan et al. [14]. This dataset provides liver mRNA expression levels of more than 45000 traits from 60 F_2 _mice generated by crossing strain C57BL/6J (B6) with BTBR. Lan et al. found that B6-ob/ob mice, when made obese, are resistant to diabetes while BEBR-ob/ob mice are not. Then the 60 animals were genotyped at 192 MIT microsatellite markers, an average of approximately 10 cM apart across the entire genome. The liver mRNA was quantified by Affymetrix M430A and B arrays. The dataset was processed using the robust multi-array average (RMA) normalization method [22]. Previous analyses of this dataset have demonstrated the increase of power by combining mapping and correlation information [12,14]. Lan et al. first used standard interval mapping [5] to map each probe at 5-cM resolution and selected 6016 " seeds", that is, gene traits with LOD score of interval mapping higher than 3.4; then 38 seeds were identified, which share the same GO term " G protein-coupled receptor" (GPCR). By combining 174 correlated traits with the 38 seeds, which are also in the GPCR protein signalling pathway, there was clear evidence of a co-regulatory region on Chr 2 at 30 cM. They also found that markers in Chr 10 may have some effects.

**Figure 4:**
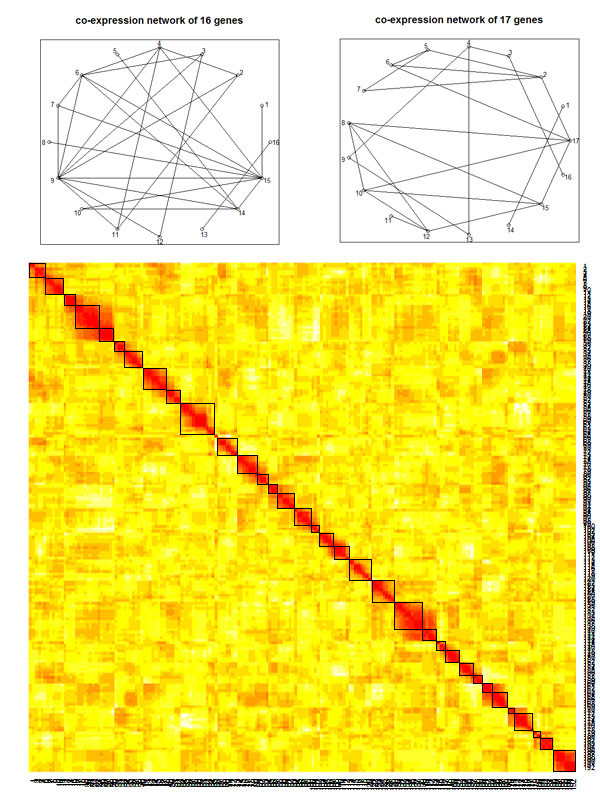
**The gene expression networks and loci LD structure**. The two co-expression networks used in the real data analysis and heat map of loci LD structure. SNPs within each chromosome are in strong LD. The marker set formation based on correlations was marked by small squares.

In our analysis, we considered two ways to form marker sets: loci within one chromosome as a marker set and loci within the boundaries where significant correlation decrease happens as a marker set. The smaller marker set formation was shown by the black squares in Figure 4. Though the adjacent loci were almost 10 cM apart, we found that SNPs located within a chromosome were still in high LD (Figure 4). For the gene expression network, it can be constructed either from the 38 seed traits or from another dataset. Under the correlation cutoff of 0.8, only 16 of the 38 traits were linked with others and used for the construction of the network (Figure 4). To mimic the practical situation with a prior network, we used the same network as Pan [12]. Using gene names, Pan identified 17 GPCR genes appearing on both our dataset and another mouse dataset with liver gene expression of 135 F2 female mice. The co-expression network was derived from the second dataset using a cutoff of 0.4 for the correlations of the 17 genes (Figure 4). For each of the 4 combinations of marker set formation and network, we applied our NGVS method and single trait selection. The big scale selection orders of the two methods and the final selection orders of the NGVS are shown in Table 2.

**Table 2:** The big scale and final selection orders of the real linkage data analysis

Network 1	Marker sets 1			Marker sets 2		
**Trt/Gene**	**Big Scale NGVS**	**Big Scale SinTrt**	**Final Selection NGVS**	**Big Scale NGVS**	**Big Scale SinTrt**	**Final Selection NGVS**

*1/Cldn4*	2, 16, 11	4, 2, 7	15, 16, 18, 161, 114	3, 27, 20	23, 5, 3	15, 16, 17, 157, 114
*2/Lor*	3, 2, 11	10, 1, 4	33, 16, 17, 18, 113	3, 6, 18	18, 14, 13	16, 17, 33, 107, 14
*3/Doc2b*	2, 6, 7	11, 5, 1	16, 18, 17, 70, 80	3, 13, 11	13, 3, 23	16, 17, 81, 68, 9
*4/F2rl2*	2, 8, 6	5, 6, 11	15, 17, 30, 93, 71	3, 14, 18	14, 3, 26	15, 17, 85, 107, 105
*5/St8sia5*	10, 18, 2	4, 6, 5	107, 106, 180, 16, 70	3, 18, 31	18, 14, 32	16, 107, 106, 180, 100
*6/Nptx1*	2, 6, 7	10, 4, 5	16, 17, 68, 80, 70	3, 6, 11	3, 13, 18	16, 17, 32, 68, 70
*7/Kcna2*	2, 6, 18	5, 1, 4	16, 68, 17, 178, 70	3, 31, 6	13, 14, 3	16, 17, 178, 180, 33
*8/Rnf17*	18, 2, 1	5, 16, 1	183, 16, 9, 180, 184	3, 32, 1	3, 10, 1	16, 17, 14, 183, 2
*9/Ankrd24*	2, 3, 11	4, 1, 2	16, 17, 33, 36, 120	3, 6, 13	13, 3, 14	16, 17, 33, 81, 119
*10/Gstm7*	2, 4, 11	5, 4, 12	16, 45, 113, 128, 17	3, 8, 27	18, 8, 3	16, 45, 17, 157, 113
*11/Tcfcp2l3*	2, 12, 6	10, 5, 7	16, 17, 122, 68, 70	3, 18, 21	18, 13, 23	16, 17, 15, 107, 122
*12/Stmn3*	3, 6, 7	7, 11, 10	36, 68, 81, 80, 32	3, 6, 13	13, 18, 3	16, 17, 32, 81, 36
*13/Rasgrf1*	13, 9, 2	4, 5, 13	131, 94, 17, 15, 96	3, 17, 22	5, 17, 8	15, 101, 131, 17, 102
*14/Sfrp1*	10, 3, 2	10, 4, 5	107, 110, 31, 16, 17	3, 18, 27	18, 3, 8	16, 107, 17, 157, 45
*15/Ergic1*	2, 3, 11	11, 4, 10	16, 17, 30, 33, 113	3, 6, 22	3, 18, 13	16, 17, 33, 131, 135
*16/Cd33*	9, 4, 13	4, 6, 5	96, 44, 131, 15, 41	8, 3, 17	14, 8, 16	44, 15, 41, 43, 101

**Network 2**	**Marker sets 1**			**Marker sets 2**		

**Trt/Gene**	**Big Scale NGVS**	**Big Scale SinTrt**	**Final Selection NGVS**	**Big Scale NGVS**	**Big Scale SinTrt**	**Final Selection NGVS**

*1/Calcrl*	9, 8, 12	4, 18, 1	101, 85, 122, 178, 121	14, 17,31	31, 2, 8	85, 101, 178, 16, 13
*2/Ccr5*	3, 2, 1	7, 1, 6	34, 15, 16, 35, 33	3, 6, 2	13, 2, 3	15, 16, 34, 10, 154
*3/Rgs6*	10, 3, 1	4, 10, 8	105, 107, 103, 37, 2	18, 23, 1	18, 9, 27	105,141, 107, 103, 106
*4/Rps6ka4*	2, 4, 12	6, 5, 9	15, 30, 16, 27, 14	3, 5, 17	5, 17, 3	15, 16, 30, 14, 28
*5/Cysltr1*	5, 3, 6	4, 5, 13	61, 57, 58, 33, 68	6, 17, 23	8, 13, 23	33, 34, 32, 100, 101
*6/P2ry12*	6, 3, 18	4, 8, 6	68, 31, 178, 40, 85	6, 31, 3	14, 25, 8	31, 178, 16, 68, 13
*7/Rassf1*	6, 3, 10	10, 11, 4	70, 68, 32, 107, 120	3, 6, 11	3, 18, 5	16, 14, 17, 32, 70
*8/Rgs3*	9, 2, 13	11, 5, 12	101, 16, 30, 26, 95	17, 3, 14	17, 3, 2	101, 16, 100, 85, 135
*9/Apln*	6, 2, 3	10, 7, 6	68, 16, 17, 70, 71	3, 11, 18	18, 13, 3	16, 68, 17, 70, 107
*10/Dok4*	15, 9, 6	15, 11, 4	154, 153, 152, 96, 68	26, 3, 17	26, 13, 5	154, 153, 152, 15, 101
*11/Lphn1*	15, 6, 8	8, 6, 10	154, 71, 156, 68, 88	3, 26, 14	14, 26, 6	16, 154, 156, 85, 17
*12/Kcnq1*	9, 15, 3	5, 3, 7	95, 157, 40, 39, 108	27, 3, 7	7, 27, 3	157, 16, 40, 13, 17
*13/Gabbr1*	4, 2, 13	4, 13, 12	44, 16, 30, 41, 45	3, 17, 14	8, 5, 3	16, 100, 85, 44, 41
*14/Gnai1*	9, 17, 6	9, 19, 17	101, 172, 170, 70, 51	17, 5, 9	17, 29,33	101, 30, 26, 27, 29
*15/Rgs3(2)*	18, 6, 12	5, 10, 1	184, 68, 70, 178, 179	31, 32,11	14, 18,17	178, 184, 68, 70, 85
*16/1200007* *D18Rik*	6, 10, 3	10, 5, 1	68, 108, 107, 109, 70	23, 3, 18	18, 23,13	140, 16, 17, 15, 107
*17/Cxcr3*	6, 13, 14	6, 11, 4	68, 131, 135, 149, 15	3, 31, 22	13, 3, 11	15, 17, 178, 131, 135

The co-expression network 1 was constructed by 16 GPCR traits and a cutoff of 0.8. The co-expression network 2 was constructed by 17 GPCR traits shared by two datasets and a cutoff of 0.4 was used. Marker set 1 and 2 denotes respectively loci within one chromosome and loci within the boundaries where significant correlation decrease happens were grouped as one markerset. Numbers in big scale orders of NGVS and Single trait selection represent the corresponding marker set numbers, while final selection orders of NGVS represent the marker numbers.

The results were consistent with Lan et al. [14]. According to the final selection order of NGVS, Marker 15, 16, 17 (*D2Mit297, D2Mit241, D2Mit9*) on Chr 2, or loci at around 30 cM, were significantly linked with GPCR genes. This region was identified as the most significant by 9 of the 16 traits in the first co-expression network under chromosome marker set scale and by all except one under smaller marker set scale. There were also weak signals that loci on Chr 10 at 40 cM have effect on the expression levels of some genes. The second co-expression network did not generate very consistent results among traits, because the 17 genes used were not highly correlated. However, we can still identify the 30 cM region on Chr 2 by 13 of the 17 genes in the top 4 selected loci with the smaller marker set division. The marker sets constructed by correlations can be treated as a more detailed division of the marker sets formed by chromosomes. It is clear that marker set formation by correlations generate more consistent and convincing results than naively setting markers within each chromosome as a group. However, in this real data case, due to the high correlations, even marker set formation by chromosomes may improve the results. Additive model and single trait selection gave specious results (not shown). The successful reproduction of existing results proved the effectiveness of NGVS.

### Kernels, interactions and covariates

In the above analysis, we only considered linear models. Covariate and epistatic effects based on different assumptions are discussed below. To simplify the analysis, we only considered a co-expression network of trait *Ankrd24, Sfrp1, Ergic1 *each connected with the other two. The following six models were formulated: (1) the additive linear model; (2) the two-effect linear model; (3) the additive linear model including adjacent interactions between markers; (4) the two-effect linear model including adjacent interactions between markers; (5) the two-effect sex-dependent model treating sex as one additional group; (6) the additive model with 5 different kernels - linear, polynomial (*q *= 2), Gaussian (*d *= 1), IBS and WIBS.

All the big scale selection orders are shown in Table 3. Ideally, we hoped the model to pick out Chr 2, 10 as the previous findings. By comparisons of Model 3, 4, 5, 6 with the basic linear model 1, 2, we made some conclusions. Under the adjacent interaction models 3 and 4, the results of additive model and two-effect model both showed improvement compared to model 1 and 2 respectively. This meant epistatic effects do have a large impact on the expression levels of mRNA. Under sex-dependent model 5, sex as one group was first identified as significant. Also, we noticed the enhancement of significance of Chr 11. The fact probably implied an underlying influence of sex on the gene expressions through loci on Chr 11. Unfortunately, the trait number was too small to draw the conclusion. Under the kernel model 6, we found that the linear kernel which simply increases the degree of freedom without changing the linear relationship of loci performed poorly; the polynomial kernel which considers all the two-way interactions together was slightly better; Gaussian, IBS, and WIBS all performed extremely well since they measure the similarities between individuals; Gaussian seemed the best way to capture the similarity as we may expect; and WIBS performed better than IBS, which proved that rare alleles indeed provide more information. From the discussion above, we notice our framework is very flexible.

**Table 3:** The first-stage selection orders of the 6 different models

Trait	Model 1 (Additive)	Model 2 (Two-effect)	Model 3 (Interactions)	Model 4 (Interactions)	Model 5 (Sex-dependent)
*Ankrd24*	6, 3, 7,18,11	2, 3,11, 4,10	10, 2, 3,13, 6	2,10, 3,11, 4	Sex,10,11, 2, 3
*Sfrp1*	6,11, 3,16,15	10, 2, 3,11, 4	10,12, 2, 3,13	10, 2,12,11,13	Sex,10,11, 4, 3
					
*Ergic1*	13, 6,15,11,12	2, 3,11,13,10	10,13, 2,12,11	2,10,13,11, 8	Sex,10, 2,11, 9

**Trait**	**Model 6** **(Kernels)**				
	
	**Linear**	**Polynomial**	**Gaussian**	**IBS**	**WIBS**

*Ankrd24*	3,18, 6,15, 7	2, 3,10,11, 5	10, 2, 1, 7,11	10, 3, 2, 6, 7	10, 2, 3, 6, 7
*Sfrp1*	11, 6, 3,15,13	10,11, 3, 2, 5	10, 2,11, 1, 7	10, 6, 3,11,12	10, 6, 3,12,11
*Ergic1*	13,15,12,11, 6	10, 2,13, 3,11	10, 2,11, 1, 7	10,13,14, 2, 6	10,13, 2,14, 9

Model 1: the additive linear model; Model 2: the two-effect linear model; Model 3: the additive linear model including adjacent interactions between markers; Model 4: the two-effect linear model including adjacent interactions between markers; Model 5: the two-effect sex-dependent model treating sex as one additional group; Model 6: the additive linear model with 5 different kernels.

In order to get general findings, we picked out marker sets Chr 2 and Chr 10, as they were identified as significant by all the 3 traits under most of the model assumptions. Then we made final selection orders for the 3 traits only using loci on Chr 2 and Chr 10. We identified marker 16, 17 (*D2Mit241, D2Mit9*) on Chr 2 and marker 107 (*D10Mit20*) on Chr 10. The region marked by *D2Mit241 *and *D2Mit9 *was obviously hot spot for those GPCR genes. Under two-effect adjacent interaction model 4 with loci on Chr 2 and 10, we went further to discover the significant epistatic effects by treating each interaction term as one variable and applying GLasso to genes in the first co-expression network. We found that 9 of the 16 gene traits exhibited the epistatic effect between marker 15 and 16 (*D2Mit297 and D2Mit241*) on Chr 2; 15 of the 16 gene traits showed the effect between marker 106 and 107 (*D10Mit148 *and *D10Mit20*) on Chr 10. It is interesting that the most significant epistatic effects occurred together with their additive and dominant effects. Under two-effect sex-dependent model 5 with loci on Chr 10 and 11, we tried to detect the sexual distinction. For Chr 10, there was no significant evidence for difference between males and females. However for Chr 11, interaction of sex with marker 118 (*D11Mit99*) was identified by 13 of the 16 gene traits. So we believe that *D11Mit99 *denotes a region which has a regulation mechanism related to sex. The results above still need further biological study.

## Conclusions

We have proposed a penalized regression method called the network-based group variable selection. The basic idea of our method is along the ongoing efforts to incorporate prior biological knowledge into data analysis. In eQTL, we hope to combine information from both the correlated gene expression traits and the loci structure [12,17,18]. By considering networks, we obtain more power to detect the co-regulatory causal SNPs; and by considering marker sets, our method gains great flexibility for modelling the complex joint activity of multiple SNPs and reduces the dimensionality of eQTL problem dramatically. We formulated the method based on these ideas and made it suitable for the efficient block co-ordinate gradient descent algorithm [20]. Furthermore, we provided the way to create the selection orders in the big and small scales and combine them together.

However, the method has some limitations. First of all, the method is designed for high-dimensional biological data such as linkage analysis or genome-wide association study, thus it is not very effective for low-dimensional selection problems. Our method is especially powerful for high-dimensional and very noisy data. In addition, combining more information means longer computation time and larger storage space. Though our method is powerful for detecting causal SNPs with moderate or weak effect, we need to try different tuning parameters λ_2 _and make λ_1 _vary with a sufficiently small step to generate the selection order. When the network is complex and the number of SNPs is large, our method is quite expensive. The storage of high-dimensional matrix is also a problem for eQTL.

We applied our method to two simulations and one real linkage dataset to demonstrate the capability of the NGVS. Simulation one compared three methods for a low-dimensional model setting and we concluded that our method is suitable for problems with high-noise background. Simulation two mimicked the real linkage data. It showed that considering the proper loci grouping, the co-expression network and the additive and dominant effects simultaneously is essential for obtaining convincing results. Under the framework of our method, we also considered many different models including kernels, interactions, and covariates in the real data analysis. All the results led to the co-regulatory regions on Chr 2, 10 for GPCR genes, which replicated the findings of Lan et al. [14]. Furthermore, we found that Gaussian kernel can depict the similarities of individuals very well; the interaction between marker *D2Mit297 *&*D2Mit241 *and between *D10Mit148 *&*D10Mit20 *are significant; and sex may have some effect on the expressions through marker *D11Mit99 *on Chr 11. Although all these conclusions need to be tested by additional research, it is clear the NGVS has the power and flexibility to handle high-dimensional problems with high-noise data successfully.

## Competing interests

The authors declare that they have no competing interests.

## Authors' contributions

WW initiated the project, invented the NGVS method, completed the simulation experiments and the linkage data analysis, and drafted the manuscript. XZ provided advice for important intellectual content and revised the manuscript. All authors read and approved the final manuscript.
